# Endoplasmic reticulum stress-mediated programmed cell death in the tumor microenvironment

**DOI:** 10.1038/s41420-025-02862-6

**Published:** 2025-12-17

**Authors:** Hongyu Chai, Qian Hu, Shun Yao, Shuoguo Ma, Wei Su

**Affiliations:** 1 Department of Gastroenterology, Digestive Disease Hospital, Affiliated Hospital of Zunyi Medical University, Zunyi, Guizhou PR China; 2 Zunyi Medical University, Zunyi, Guizhou PR China

**Keywords:** Cancer microenvironment, Physiology

## Abstract

Endoplasmic reticulum stress (ERS) dynamically regulates cell fate decisions within the tumor microenvironment (TME) through the PERK, IRE1α, and ATF6 pathways of the unfolded protein response (UPR), forming an “ERS-Death Axis” interconnected with apoptosis, autophagy, pyroptosis, and ferroptosis. Its molecular network involves CHOP-mediated apoptotic imbalance, NLRP3 inflammasome-activated pyroptosis, the ATF4–CHAC1 axis-driven ferroptosis, and the dual roles of autophagy (protective or pro-death). Oxidative stress further amplifies the biological functions of this network. The ERS-Death Axis exhibits significant heterogeneity across different tumors. Therapeutic strategies targeting this axis have demonstrated clear potential, including specific modulation of core UPR molecules, pathway activation by natural compounds, synergistic combinations with immune checkpoint inhibitors and metabolic interventions, and enhanced targeting and efficacy through nanodelivery systems. However, clinical translation faces key challenges such as tumor heterogeneity, drug delivery efficiency, and complex resistance mechanisms. In-depth elucidation of the tumor-specific mechanisms underlying the ERS-Death Axis will provide crucial theoretical support for overcoming bottlenecks in cancer therapy and optimizing combination treatment regimens, propelling this axis to become a core target for precision oncology.

## Facts

- ERS regulates cell death modalities such as apoptosis and autophagy through the UPR pathway (PERK, IRE1α, ATF6), forming an “ERS–Death Axis” that is closely associated with the tumor microenvironment.
- Hypoxia, nutrient deprivation, and oxidative stress are major drivers of ERS in the tumor microenvironment, influencing tumor progression and therapy resistance.
- The ERS–Death Axis exhibits specificity across different tumors (e.g., breast cancer, colorectal cancer, liver cancer), involving distinct molecular mechanisms and regulatory pathways.
- Therapeutic strategies targeting core UPR pathways and downstream molecules (e.g., CHOP, GRP78) show anti-tumor potential in preclinical studies, and combination therapies (e.g., with immunotherapy or metabolic intervention) can enhance efficacy.

## Open questions

- How can personalized treatment standards be developed based on tumor subtype-specific features of the ERS–Death Axis?
- How can the activation intensity of ERS be precisely regulated to balance antitumor effects and toxicity to normal tissues?
- How can ERS-mediated drug resistance mechanisms (e.g., exosome-mediated escape) be effectively blocked?

## Introduction

The endoplasmic reticulum (ER), a central organelle responsible for protein folding and calcium homeostasis, undergoes functional disruption that triggers endoplasmic reticulum stress (ERS)—a prevalent stress response in the tumor microenvironment (TME) [1, 2]. Under TME pressures such as hypoxia, nutrient deprivation, and oxidative stress, tumor cells accumulate excessive unfolded or misfolded proteins in the ER lumen, thereby activating the unfolded protein response (UPR). This response initiates adaptive regulation through three core pathways—PERK, IRE1α, and ATF6—to sustain cell survival [3]. However, when ERS persists or becomes overactivated, it surpasses cellular adaptive thresholds and subsequently modulates various forms of programmed cell death (PCD), including apoptosis, autophagy, pyroptosis, and ferroptosis, thereby contributing to tumorigenesis, therapy resistance, and immune microenvironment remodeling [4, 5].

The crosstalk network between ERS and PCD (termed the “ERS-Death Axis”) exhibits high complexity. On one hand, ERS can induce apoptosis via CHOP-mediated Bcl-2 family imbalance and JNK pathway activation [6–9], trigger pyroptosis through NLRP3 inflammasome activation [10], or promote ferroptosis via the ATF4–CHAC1 axis [11, 12]. On the other hand, ERS-induced autophagy may either exert a protective role by clearing misfolded proteins or switch to a pro-death effect under lysosomal dysfunction [13, 14]. This bidirectional regulation is influenced by tumor type, microenvironmental conditions, and genetic background, as evidenced by the heterogeneous ERS responses across different breast cancer subtypes [15, 16].

A comprehensive understanding of the molecular mechanisms underlying the ERS-Death Axis, along with the identification of its tumor-specific regulatory patterns, is crucial for developing precision therapeutic strategies targeting ERS. This review systematically examines the interplay between ERS and various PCD pathways, the amplifying role of oxidative stress, and immune microenvironment modulation, while summarizing the distinct features of the ERS-Death Axis across tumor types. Furthermore, we discuss its clinical translation potential and challenges, aiming to provide novel theoretical insights and intervention targets for cancer therapy.

## Driving factors of ERS in the TME

**Fig. 1:**
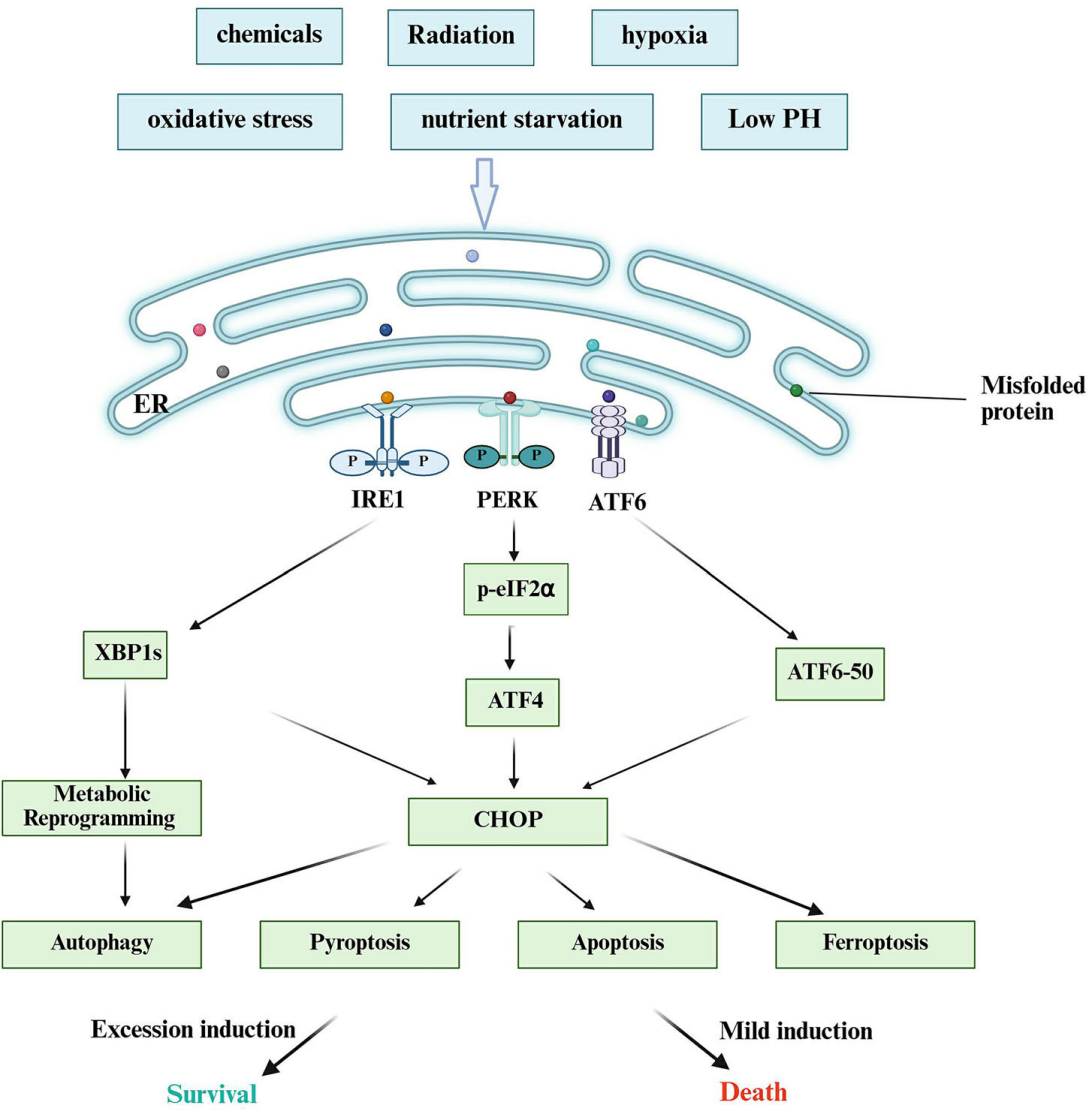
Mechanisms of unfolded protein response (UPR) pathways in determining cell fate under tumor microenvironment (TME) stress. Multiple stress conditions within the TME lead to the accumulation of unfolded/misfolded proteins in the endoplasmic reticulum (ER), triggering ER stress (ERS). This activates three key UPR sensors located on the ER membrane: PERK, IRE1α, and ATF6. The PERK–eIF2α–ATF4 axis primarily induces the expression of the transcription factor CHOP (C/EBP homologous protein), which serves as a central mediator of apoptosis and pyroptosis while also modulating genes involved in ferroptosis. Simultaneously, the IRE1α–XBP1s axis activates adaptive responses through metabolic reprogramming, such as enhanced lipogenesis, and protective autophagy to alleviate cellular stress. The ATF6 pathway contributes to cellular adaptation by upregulating ER chaperone proteins including GRP78 and participates in CHOP-mediated apoptotic signaling. Integration of these signaling cascades, particularly through the molecular hub CHOP, determines the ultimate cellular fate: promoting survival under mild or transient stress conditions, or driving cell death through diverse programmed cell death modalities under severe or prolonged stress. CHOP thus functions as a critical molecular switch balancing pro-survival and pro-death outcomes in response to proteotoxic stress. Created with BioRender.com.

The drivers of ERS in the TME constitute a complex pathophysiological process involving the synergistic effects of multiple microenvironmental stressors. The rapid proliferation of tumor tissue leads to significant alterations in the local microenvironment, including hypoxia, nutrient deprivation, oxidative stress, and acidosis—harsh conditions that collectively serve as primary inducers of ERS [5] (Fig. 1). Hypoxia, a hallmark feature of the TME, not only directly disrupts ER protein folding by inhibiting disulfide bond formation but also upregulates the expression of redox enzymes such as ERO1α through HIF-1α activation, thereby influencing tumor angiogenesis and immune evasion [17, 18]. This hypoxia-induced ERS plays a pivotal role in tumor progression and therapy resistance, particularly in modulating chemosensitivity and immunotherapy response [19, 20].

Nutrient deprivation, another critical characteristic of the TME, triggers ERS through multiple mechanisms [5]. The high demand for nutrients by rapidly proliferating tumor cells leads to deficiencies in essential metabolites such as glucose and amino acids, activating the IRE1α/XBP1 signaling pathway to promote lipid synthesis and ER expansion. This metabolic reprogramming not only sustains tumor cell survival but also provides alternative energy sources by regulating autophagy [21–23]. Notably, nutrient deprivation-induced ERS also influences the tumor immune microenvironment, transmitting regulatory signals via exosomes and other carriers to suppress T-cell function and promote the polarization of immunosuppressive M2-type macrophages [24].

External therapeutic pressures, including chemotherapy and radiotherapy, are also significant contributors to ERS in tumor cells [25]. Certain targeted therapies, such as immune checkpoint inhibitors (ICIs), may enhance tumor cell immune evasion by upregulating ERS markers like GRP78 [23], highlighting the dual role of ERS in therapy resistance. Therefore, intervention strategies targeting ERS pathways—such as combining ERS inducers with immunotherapies—may represent a novel approach to overcoming treatment resistance.

## Core mechanisms of ERS-mediated tumor regulatory networks

### The interactive network between ERS and programmed cell death

#### The ERS-apoptosis axis

Apoptosis, as a crucial form of PCD, plays a central role in maintaining tissue homeostasis and organismal development [26, 27]. This highly conserved physiological process is activated through two major pathways—the intrinsic (mitochondrial) and extrinsic (death receptor) pathways—and is characterized by cellular shrinkage, chromatin condensation, DNA fragmentation, and apoptotic body formation [28–30]. In tumor biology, dysregulation of apoptotic pathways has been widely recognized as a hallmark of cancer, with its abnormalities not only promoting tumorigenesis and progression but also closely associated with chemotherapy resistance [31].

**Fig. 2:**
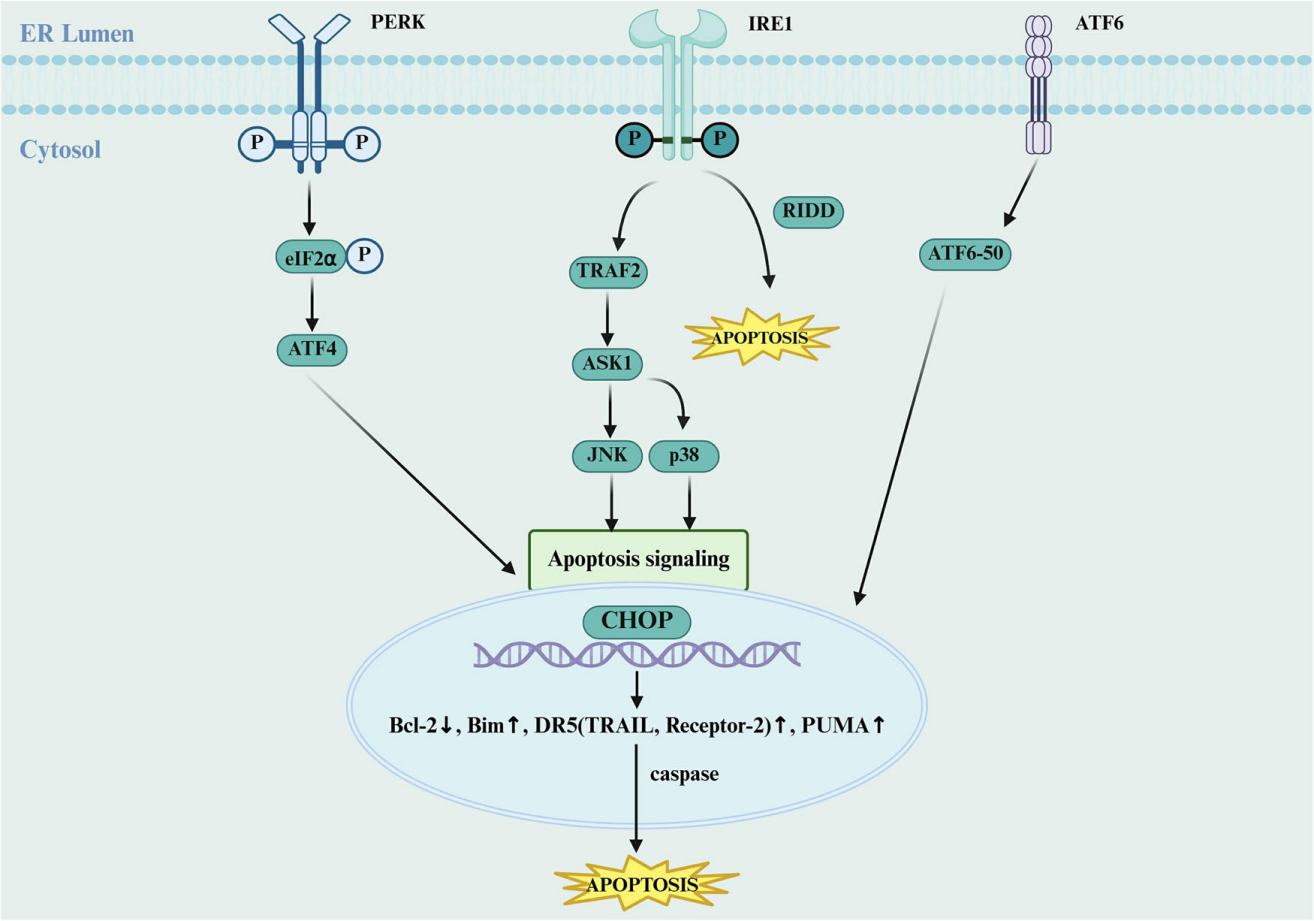
ERS-induced apoptosis. ERS triggers the molecular cascade of apoptosis through the PERK, IRE1, and ATF6 pathways. Under ERS conditions, the IRE1 pathway is activated through the kinase domain of IRE1, which recruits and binds TRAF2 to initiate the ASK1-JNK/p38 MAPK signaling cascade. This leads to phosphorylation of Bcl-2 (inhibiting its anti-apoptotic function) and activation of the pro-apoptotic protein Bim. Simultaneously, IRE1 degrades survival factor mRNAs via the RIDD mechanism, collectively promoting apoptosis. In the PERK pathway, ERS induces PERK-mediated phosphorylation of eIF2α, enabling selective translation of ATF4. ATF4 then translocates to the nucleus and upregulates CHOP expression, which promotes pro-apoptotic molecules such as Bim and PUMA while suppressing Bcl-2, ultimately initiating the caspase cascade. The ATF6 pathway involves its proteolytic cleavage (generating ATF6-50), which regulates CHOP and death receptor DR5 (TRAIL Receptor-2) expression, thereby activating the caspase-8-dependent extrinsic apoptotic pathway. These three pathways are not independent but exhibit crosstalk and synergistic regulation through key nodes such as CHOP and apoptosis-related proteins, collectively determining the cellular fate between survival and apoptosis under ERS conditions. ER endoplasmic reticulum; PERK protein kinase RNA-like endoplasmic reticulum kinase, IRE1 inositol requiring enzyme 1, AFT6 transcription factor 6. Created with BioRender.com.

The crosstalk between ERS and apoptosis constitutes a critical molecular network in the TME. At the core of this network lies the UPR system, which senses and responds to proteostatic imbalance in the ER through three primary pathways (IRE1, PERK, and ATF6), exhibiting highly pathway-specific regulatory mechanisms (Fig. 2) [32, 33]. Adaptive ERS helps modulate protein synthesis to maintain cellular homeostasis [34]. However, under the sustained ERS conditions of the TME, the UPR system shifts from a pro-survival to a pro-apoptotic mode, a transition involving a cascade of key molecular events [35, 36]. Notably, the expression level of CHOP (C/EBP homologous protein, also known as GADD153) is frequently used as a critical biomarker for assessing ERS-induced apoptosis [37–39]. The upregulation of the CHOP transcription factor represents a central event in this process, initiating the caspase cascade by regulating the balance of the Bcl-2 family (promoting BIM/PUMA while suppressing Bcl-2), ultimately triggering apoptosis [6].

In recent years, ERS-induced apoptotic pathways have demonstrated significant clinical potential in cancer therapy. Multiple studies have shown that targeted modulation of ERS-related pathways can effectively induce tumor cell apoptosis while overcoming resistance to conventional treatments. The Fujian Wan team discovered that knockout of the YTHDF2 gene enhances ERS through the GLI2–JNK pathway, significantly suppressing stemness traits and promoting apoptosis in cervical cancer cells, offering a novel therapeutic target [40]. Diterpenoid tanshinone exhibits potent antitumor effects in lung adenocarcinoma models via the IRE1α/caspase12 pathway [41]. Particularly noteworthy is the synergistic combination of 2-Deoxyglucose and hydroxychloroquine, which induces apoptosis in triple-negative breast cancer through the PERK–ATF4–CHOP axis while simultaneously inhibiting protective autophagy [42]. In drug delivery systems, ER-targeted nanoparticles encapsulating siGRP94 have been developed for hepatocellular carcinoma (HCC), triggering ERS cascades and apoptosis through forced calcium influx, achieving tumor-specific therapy [43].

#### The ERS-autophagy axis

**Fig. 3:**
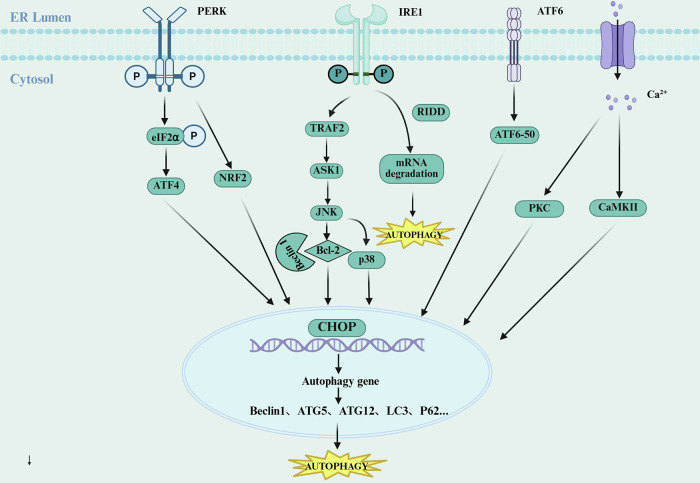
ERS-induced autophagy. The IRE1α pathway plays a central regulatory role in the molecular mechanism of the ERS-autophagy axis through its dual functionality. On one hand, its kinase domain activates the JNK signaling pathway, promoting the dissociation of the Bcl-2/Beclin1 complex and initiating autophagosome formation. On the other hand, its nuclease activity selectively degrades mRNAs encoding mitochondrial quality control proteins (such as PINK1) via the RIDD mechanism, thereby regulating selective autophagy. In the PERK-eIF2α-ATF4 pathway, ERS activates PERK, which phosphorylates eIF2α to inhibit global protein synthesis while selectively translating ATF4. As a transcription factor, ATF4 regulates the expression of a series of autophagy-related genes. For instance, it induces CHOP expression, which further promotes the transcription of autophagy-related genes. Additionally, ATF4 directly binds to the promoter regions of autophagy-related genes, enhancing the expression of key autophagy molecules such as Beclin1, ATG5, ATG12, and LC3, thereby inducing autophagy. In the ATF6 pathway, upon ERS activation, ATF6 translocates from the ER to the Golgi apparatus, where it is cleaved and activated. The released cytosolic domain then enters the nucleus to regulate autophagy-related genes. Furthermore, ERS disrupts ER calcium homeostasis, leading to the release of calcium ions into the cytoplasm. This activates calcium-dependent autophagy pathways via PKC and CaMKII signaling. Created with BioRender.com.

The interplay between ERS and autophagy constitutes a sophisticated regulatory network that plays a dual role in tumorigenesis and progression. As a crucial homeostatic mechanism, autophagy is markedly activated under ERS conditions, forming a dynamic ERS-autophagy regulatory axis [44]. Similar to the ERS-apoptosis axis, this process is primarily regulated through the three canonical UPR pathways (Fig. 3), yet exhibits distinct molecular signatures and biological consequences [45]. During the initial phase of ERS, acute stress predominantly triggers protective autophagy, facilitating the clearance of misfolded proteins and damaged organelles to maintain cellular viability [46]. However, under persistent ERS conditions, chronic stress leads to impaired autophagic flux and lysosomal dysfunction, potentially switching autophagy toward a pro-death function [13, 14]. This precisely orchestrated network enables cells to mount context-dependent responses based on stress intensity and duration, ultimately determining cellular fate [47].

The UPR-mediated activation of autophagy has been extensively validated across various tumor models. In HepG2 hepatoblastoma cells, hydrogen peroxide-induced oxidative damage is potentiated by the ERS inducer tunicamycin, accompanied by increased LC3II/LC3-I ratio and p62 degradation - effects that are reversed by the ERS inhibitor salubrinal, unequivocally demonstrating direct ERS-autophagy crosstalk [48]. Colorectal cancer (CRC) studies reveal that excessive proliferation-induced ERS activates protective autophagy through IRE1α and PERK pathways, promoting tumor cell survival and drug resistance [49]. These findings not only elucidate resistance mechanisms but also inform novel combination therapies. Therapeutic modulation of the ERS-autophagy axis has shown promising antitumor efficacy. Sequential treatment strategies - initiating UPR with ERS inducers followed by autophagy inhibitors - demonstrate remarkable synergy in refractory cancers like triple-negative breast cancer [42]. Notably, carbon monoxide-induced moderate ERS activates protective autophagy in T cells, enhancing their antitumor functionality and suggesting novel immunotherapeutic approaches [50]. Furthermore, prognostic models incorporating ERS and mitophagy-related genes exhibit robust predictive value in lung adenocarcinoma, highlighting their clinical potential [51].

The ERS-autophagy interaction displays remarkable tissue specificity. In ovarian cancer, ERS suppresses PI3K/AKT/mTOR signaling to concurrently promote autophagy and apoptosis, increasing chemosensitivity [52]; whereas in photodynamic therapy for cholangiocarcinoma, ERS-induced autophagy initially exerts cytoprotection but transitions to pro-death effects upon lysosomal damage [53]. Such context-dependence underscores the need for personalized therapeutic approaches. Mechanistically, emerging epigenetic regulators like the LINC00963/miR-320a/XBP1 axis [54] and ZNF263-mediated super-enhancer activation [55] provide novel molecular insights into tumor heterogeneity and targeted intervention.

#### The ERS-pyroptosis axis

**Fig. 4:**
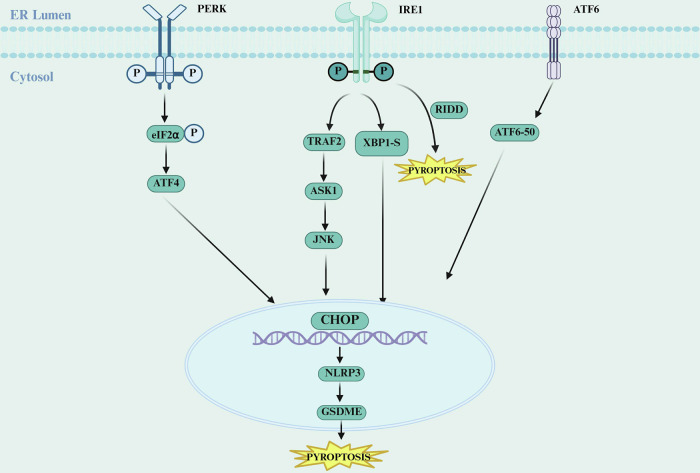
ERS-induced pyroptosis. In tumors, the ERS-pyroptosis axis involves three core signaling pathways that interconnect ERS and pyroptosis. Activated IRE1α initiates the downstream JNK signaling pathway through its kinase domain, leading to phosphorylation and activation of transcription factors that regulate pyroptosis-related gene expression. Concurrently, its ribonuclease activity modulates mRNA stability via the RIDD pathway, altering the intracellular protein profile and further contributing to pyroptosis regulation. ERS also activates PERK, which phosphorylates eIF2α to selectively enhance ATF4 translation. Upregulated ATF4 increases CHOP expression, which in turn promotes pyroptosis by facilitating the interaction between TXNIP and NLRP3, thereby activating the NLRP3 inflammasome. Additionally, CHOP impairs mitochondrial function, causing mitochondrial damage and the release of mitochondrial components such as mitochondrial DNA, which further amplifies NLRP3 inflammasome activation and drives pyroptosis. The ATF6 pathway modulates pyroptosis indirectly by downregulating P2×7R/NLRP3 or activating sFRP2/NF-κB signaling. Proteolytic cleavage of ATF6 can also trigger caspase-4-dependent NLRP3 inflammasome activation. These collective mechanisms ultimately lead to cleavage of GSDMD and maturation and release of IL-1β/IL-18, resulting in pyroptotic cell death. Created with BioRender.com.

Pyroptosis, as a novel form of programmed inflammatory cell death, plays a critical role in infections, inflammatory diseases, and tumor immunity [56, 57]. It is characterized by the formation of pores in the cell membrane, leading to the release of cellular contents and triggering strong inflammatory responses. This process is mediated by key effector proteins of the Gasdermin family, particularly Gasdermin D (GSDMD) and Gasdermin E (GSDME) [58–60]. Within the TME, the dual regulatory role of pyroptosis has attracted increasing attention, as it can not only activate anti-tumor immunity by releasing damage-associated molecular patterns (DAMPs) but may also promote inflammation-associated tumor progression [61, 62]. The molecular coupling between ERS and pyroptosis forms a complex regulatory network, with its core mechanism lying in the interaction between the UPR and NLRP3 inflammasome activation [10] (Fig. 4).

The ERS-pyroptosis axis exhibits dual effects in the TME. On one hand, it can induce immunogenic cell death (ICD) in tumor cells, releasing DAMPs such as ATP, HMGB1, and calreticulin, which promote dendritic cell (DC) maturation and T cell activation, thereby enhancing anti-tumor immune responses [56]. For example, the metabolite trimethylamine N-oxide induces tumor cell pyroptosis by activating the PERK pathway, improving the response to immunotherapy in triple-negative breast cancer [63]. Nanoparticles such as CS-HAP@KAE can convert “cold” tumors into “hot” tumors through the ERS-pyroptosis axis, enhancing the efficacy of ICIs [64]. On the other hand, excessive activation of the ERS-pyroptosis axis may exacerbate tumor-associated inflammation and immune suppression. For instance, in renal cell carcinoma, STING inhibits ERS-mediated pyroptosis to promote immune evasion, while targeted degradation of STING restores PERK/CHOP-dependent pyroptosis and enhances anti-tumor immunity [65]. Additionally, the ERS-pyroptosis axis is closely associated with metabolic reprogramming, such as glucose metabolism disorders and TXNIP/NLRP3 pathway activation, influencing tumor progression [66, 67]. For example, the ZDHHC1 gene suppresses tumor growth by inducing ERS-pyroptosis, while ginsenosides and flavonoids inhibit pyroptosis in diabetes-associated tumors by modulating the ERS-TXNIP/NLRP3 axis [68].

In terms of therapeutic strategies, targeting the ERS-pyroptosis axis has emerged as a promising direction. For instance, baicalin reduces pyroptosis in Mycobacterium tuberculosis-infected macrophages by inhibiting the PERK/TXNIP/NLRP3 pathway [69]. Nanoplatforms such as PCAN enhance anti-tumor immunity by specifically inducing ERS-dependent type II ICD and pyroptosis [70].

#### ERS-ferroptosis axis

**Fig. 5:**
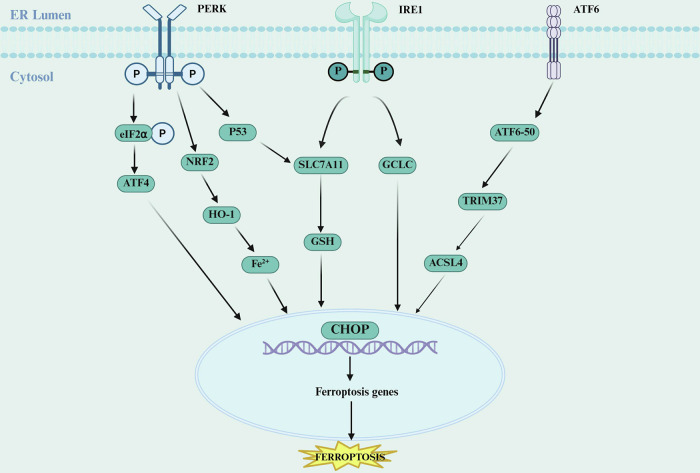
ERS-induced ferroptosis. The PERK-eIF2α-ATF4 signaling axis plays a critical role in ERS-mediated ferroptosis. Activation of PERK leads to phosphorylation of eIF2α, which promotes the translation of ATF4. This in turn upregulates pro-ferroptotic genes such as CHOP and CHAC1. CHAC1 degrades glutathione, thereby weakening the cellular antioxidant capacity, while ATF4 may also modulate HO-1 to increase the labile iron pool, promoting the Fenton reaction and lipid peroxidation. The IRE1α-XBP1 pathway exhibits a dual role in ferroptosis regulation. On one hand, the RNase activity of IRE1α can cleave and downregulate genes involved in glutathione synthesis (e.g., SLC7A11), reducing antioxidant defense and facilitating ferroptosis. On the other hand, IRE1α-XBP1 signaling may indirectly suppress ferroptosis by maintaining endoplasmic reticulum homeostasis. The role of the ATF6 pathway in ferroptosis is relatively less studied, but emerging evidence suggests that ATF6 can transcriptionally regulate TRIM37 to influence ferroptosis. In cervical cancer, ATF6 activation induces TRIM37 expression, which ubiquitinates and degrades ACSL4—a key enzyme promoting lipid peroxidation—thereby inhibiting ferroptosis. Furthermore, the crosstalk between ERS and iron metabolism represents another crucial mechanism in ferroptosis regulation. ERS can promote intracellular iron accumulation through calcium signaling (e.g., the SLC12A5-PNCK pathway) or via upregulation of transferrin. Created with BioRender.com.

Ferroptosis is a unique mode of cell death driven by iron-dependent phospholipid peroxidation, involving dysregulated iron metabolism, imbalance in the lipid antioxidant system, and accumulation of lipid peroxides [71]. The high metabolic demands and hypoxic microenvironment created by the malignant proliferation of tumor cells can lead to the accumulation of misfolded or unfolded proteins in the ER, thereby triggering ERS [71]. These two processes interact through core signaling pathways such as PERK-eIF2α-ATF4, IRE1α, and ATF6 (Fig. 5).

The ERS-ferroptosis axis exhibits a dual role in tumors. On one hand, it provides new therapeutic targets. Tanshinone IIA can exert anti-tumor effects by downregulating the PERK–ATF4–HSPA5 pathway-mediated activation of ferroptosis [72]. The irreversible proteasome inhibitor carfilzomib, combined with ^125^I brachytherapy, synergistically induces apoptosis, necroptosis, and ferroptosis by enhancing ERS and the UPR, producing significant anti-tumor effects in esophageal squamous cell carcinoma [73]. Nanodrug delivery systems, such as pH-sensitive nanoplatforms [74] and iron-based metal-organic framework nanocages [75], can achieve tumor-specific therapy by inducing ferroptosis. Combining the regulation of the ERS-ferroptosis axis with ICIs to induce ferroptosis in tumor cells, or the synergistic effect of proteasome inhibitors with ferroptosis signaling suppression, offers new strategies to enhance anti-tumor efficacy [76, 77]. On the other hand, tumor cells may exploit this axis to evade death [78]. In non-small cell lung cancer, GPER1 can promote the activation of PI3K/AKT/mTOR signaling, induce SCD1 expression, and thereby prevent ferroptosis in tumor cells, facilitating tumor progression [79]. The TME components, such as immune cells and extracellular matrix, engage in complex signaling crosstalk with the ERS-ferroptosis axis.

#### CHOP: a central integrator of ERS-mediated cell death

CHOP belongs to the CCAAT/enhancer-binding protein (C/EBPs) family. It is a 29 kD protein consisting of 169 amino acid residues in humans (168 in rodents). Its structure comprises two functional domains: an N-terminal transcriptional activation domain and a C-terminal basic leucine zipper (bZIP) domain [35]. Under normal physiological conditions, CHOP is ubiquitously expressed at very low levels [80]. However, under persistent ERS conditions, it is significantly transcriptionally upregulated through all three branches of the UPR—PERK–eIF2α–ATF4, IRE1α–XBP1, and ATF6—making it a reliable marker of severe ERS [3].

As a core effector downstream of ERS, CHOP orchestrates multiple forms of PCD by regulating downstream target genes. In apoptosis, CHOP initiates the caspase cascade by altering the balance of Bcl-2 family proteins (promoting BIM/PUMA expression and suppressing Bcl-2) [6–9]; in autophagy, it modulates autophagic flux through the transcriptional regulation of key autophagy-related genes [44]; in pyroptosis, CHOP interacts with inflammatory pathways such as the NLRP3 inflammasome, participating in the regulation of PERK/CHOP-dependent pyroptosis [10, 65]; in ferroptosis, it promotes iron-dependent cell death by regulating genes involved in redox and iron metabolism (e.g., CHAC1 and SAT1) [11, 12]. Notably, as a member of the C/EBPs family, CHOP itself is involved in regulating genes encoding proteins related to proliferation, differentiation, and energy metabolism, playing an important role in determining cell fate [71].

The central regulatory role of CHOP in these death pathways establishes its function as a molecular “switch” that determines cell survival or death under stress conditions. This multifaceted regulatory capability highlights the important therapeutic significance of CHOP in targeting ERS-driven cell death in cancer. For example, the synergistic combination of 2-deoxyglucose and hydroxychloroquine induces apoptosis in triple-negative breast cancer cells via the PERK–ATF4–CHOP axis [42], while targeted degradation of STING enhances antitumor immunity by restoring PERK/CHOP-dependent pyroptosis [65]. These findings indicate that CHOP is not only a key biomarker of ERS but also a promising target for therapeutic intervention, providing an important theoretical basis for developing novel anticancer strategies.

### The amplifier role of oxidative stress

Oxidative stress and ERS exhibit a tightly coordinated “amplification” relationship during tumorigenesis and progression. As a key driver, oxidative stress can exacerbate ERS through multiple pathways, thereby reshaping the biological behavior of tumor cells and the TME. The large amounts of reactive oxygen species (ROS) generated by oxidative stress directly disrupt the redox homeostasis of the ER, leading to protein misfolding and aggregation—a core trigger of ERS. For example, in glioblastoma, glutamine deficiency reduces glutathione levels, significantly elevating ROS and causing the accumulation of unfolded proteins in the ER lumen, which activates ERS sensor proteins such as PERK, IRE1α, and ATF6, initiating the UPR [81]. Meanwhile, the activation of ERS signaling pathways can, in turn, promote ROS generation, forming a vicious cycle. For instance, oridonin-induced ERS in CRC cells disrupts ER calcium homeostasis, releasing calcium ions into the cytoplasm and activating mitochondrial ROS production, further intensifying oxidative stress and driving tumor cells toward death [82].

The “amplification” regulation of ERS-related signaling pathways by oxidative stress profoundly influences tumor cell metabolic reprogramming and survival adaptability. In M2 tumor-associated macrophages, oxidative stress synergistically activates ERS via the IRE1-XBP1 pathway, suppressing glycolysis while promoting oxidative phosphorylation and lipid accumulation, thereby maintaining the immunosuppressive M2 phenotype and fostering a favorable environment for tumor growth [83]. In cancer cells, sustained oxidative stress-ERS conditions drive metabolic shifts that favor tumor proliferation, such as upregulating key glycolytic enzymes to enhance glucose uptake and utilization while inhibiting fatty acid oxidation to ensure biosynthetic precursor supply [84, 85]. Moreover, the mutual amplification of oxidative stress and ERS significantly affects tumor cell sensitivity and resistance to therapy. In chemodynamic therapy, H₂O₂ self-supplying nanocomposites (e.g., (Cu₂SeCa₂) @LA) generate ROS under near-infrared light, triggering ERS and upregulating the PERK-mediated eIF2α phosphorylation pathway to induce ICD, thereby killing tumors [85]. However, tumor cells can also exploit the oxidative stress-ERS interplay to develop resistance—for example, by activating the ER chaperone protein GRP78 to repair misfolded proteins while enhancing the antioxidant defense system to reduce ROS levels and diminish therapeutic efficacy [86]. Additionally, ERS induced by oxidative stress can release DAMPs, such as calreticulin and high-mobility group box 1 (HMGB1), activating dendritic cells and modulating the TME to influence immunotherapy outcomes [87–89].

### Regulation of the immune microenvironment

The ERS in the TME plays a pivotal role in tumor immune escape by modulating immune cell functions. Research indicates that the UPR induced by ERS exerts dual regulatory effects in both tumor cells and immune cells—sustaining tumor cell survival while also reshaping the immune microenvironment through non-cell-autonomous mechanisms. In tumor cells, activation of key UPR molecules such as the IRE1α/XBP1 and PERK/ATF4 pathways promotes the formation of an immunosuppressive TME. For instance, XBP1 upregulates cholesterol synthesis and secretion, activating myeloid-derived suppressor cells and thereby inhibiting CD8(+)T cell function [76, 90]. Additionally, ERO1A influences immunogenic cell death in tumor cells by regulating the balance between IRE1α and PERK signaling, attenuating the therapeutic efficacy of PD-1 blockade [91]. Clinical data further reveal that lung cancer patients with high ERO1A expression exhibit poor responses to neoadjuvant immunotherapy, suggesting its potential as a biomarker for immunotherapy resistance [91].

ERS also modulates immune checkpoint molecule expression to affect T cell function. For example, metformin activates AMPK to phosphorylate PD-L1 at the S195 site, promoting its degradation via the ER-associated degradation pathway and thereby enhancing cytotoxic T lymphocyte activity [92]. Similarly, ERS-induced ATF4 can deliver the long non-coding RNA SNHG6 through small extracellular vesicles, suppressing T cell-mediated immune responses and promoting M2 macrophage polarization to establish an immunosuppressive TME [93]. In HCC, ERS-related gene signatures significantly correlate with immune cell infiltration and poor prognosis, with low GP6 expression strongly linked to immunotherapy resistance and reduced survival rates [94].

ERS exerts particularly notable effects on myeloid cell function. In glioblastoma, IRE1α recruits monocytes and neutrophils via the UBE2D3/NF-κB axis, fostering a pro-tumor immune microenvironment [95]. Moreover, the PERK pathway upregulates phosphoserine aminotransferase 1 and serine metabolism to enhance the immunosuppressive function of M2 macrophages, while PERK inhibition improves the efficacy of PD-1 blockade [96]. In melanoma models, the PERK inhibitor GSK2656157 combined with an mRNA vaccine promotes M1 macrophage polarization and enhances CD8(+) T cell infiltration, significantly suppressing tumor growth and metastasis [97]. ERS also influences antitumor immune responses by regulating ICD. For instance, the photosensitizer ER-Cy triggers NLRP3 inflammasome activation and pyroptosis via ERS induction, releasing DAMPs to promote dendritic cell maturation and T cell activation [98]. Similarly, nanoparticle-mediated ERS activation enhances the ICD effect of chemotherapeutic drugs by releasing ATP and HMGB1, which activate antigen-presenting cells and reverse the immunosuppressive TME [99].

## Characteristics of the ERS-death axis in different tumor types

### Breast cancer (BC)

BC exhibits a multi-layered and dynamically evolving interaction between ERS and cell death. At the molecular level, ERS regulates cell fate through three major signaling pathways, with the PERK/eIF2α/ATF4/CHOP pathway playing a pivotal role in determining cell survival or death [100]. Notably, different BC subtypes display significant heterogeneity in ERS responses: in estrogen receptor-positive (ER+) breast cancer, moderate ERS activation promotes cellular adaptation, whereas sustained activation induces apoptosis through mitochondria-associated endoplasmic reticulum membrane-mediated calcium signaling and ROS production [15, 16]. In contrast, triple-negative breast cancer, which exhibits higher basal ERS levels, is more sensitive to ERS inducers but also develops resistance mechanisms through anti-apoptotic proteins such as FLIP [101].

At the TME level, stress factors such as hypoxia, acidosis, and nutrient deprivation persistently activate the UPR, while ERS, in turn, remodels the TME [102, 103]. This bidirectional regulation manifests in two ways: on one hand, ERS induces ICD through calreticulin exposure, HMGB1 release, and ATP secretion, enhancing anti-tumor immunity [104]; on the other hand, it promotes immune suppression via mechanisms such as PD-L1 glycosylation [103]. Particularly noteworthy is the discovery that ERS-induced exosomal miR-27a-3p upregulates macrophage PD-L1 expression through the MAGI2/PTEN/PI3K axis [103], providing a novel perspective for understanding tumor immune escape. In terms of therapeutic translation, ERS-targeting strategies can be broadly categorized into two approaches: first, direct modulation of core ERS molecules, such as combining the PERK agonist CCT020312 with taxanes to overcome drug resistance [105, 106]; second, the use of natural compounds (e.g., γ- and δ-tocotrienols [107], oleandrin [104]) to specifically activate the ERS-death axis.

### Colorectal cancer (CRC)

The interaction between ERS and cell death in CRC exhibits complex and dynamic regulatory characteristics. Studies have shown that the ferroptosis inducer RSL3 significantly activates all three major pathways of the UPR, with the PERK pathway negatively regulating ferroptosis by modulating ATF4’s binding ability to the SLC7A11 promoter [108]. This discovery highlights the critical role of ERS pathways in regulating ferroptosis sensitivity, offering a novel approach to overcoming apoptosis resistance.

In terms of the interplay between ERS and the immune microenvironment, research has revealed that ERS-induced ICD exhibits dual regulatory properties. Macrocarpal I, by activating the PERK/eIF2α/ATF4/CHOP pathway, not only triggers classic ICD markers such as calreticulin exposure and HMGB1 release but also induces ferroptosis. This dual-death mechanism significantly enhances the immune response to anti-PD-1 therapy [109]. However, sustained ERS may also promote immune escape through exosomal miR-27a-mediated PD-L1 upregulation [110], underscoring the importance of temporally controlled therapeutic strategies.

Various ERS-targeting treatment approaches have shown promising translational potential. Natural compounds such as curcumin and gambogenic acid induce ERS-dependent apoptosis by specifically activating the ATF6 and IRE1α pathways [111, 112], while α-hederin causes irreversible proteostasis collapse by simultaneously blocking ERAD and autophagic flux [113]. Notably, cetuximab-resistant cells exhibit sensitivity to the proteasome inhibitor carfilzomib [114], revealing a new strategy for overcoming targeted therapy resistance through ERS modulation. Additionally, the SPARC protein competitively binds to GRP78, relieving its anti-apoptotic effects and providing a novel approach to restoring chemotherapy sensitivity [115].

### Hepatocellular carcinoma (HCC)

In HCC, the regulation of the ERS-death axis primarily revolves around the three core UPR pathways, influencing tumor progression and therapeutic response through apoptosis, autophagy, and specialized forms of cell death. Various natural compounds and drugs can induce HCC cell death by activating ERS pathways. For instance, Icaritin triggers ERS and mitochondrial dysfunction by targeting BHLHE40, leading to caspase-independent cytoplasmic vacuolization [116]. Fisetin induces intracellular Ca²⁺ release, activating the PERK–ATF4–CHOP pathway while promoting GRP78 exosome secretion. It also enhances ERS-mediated apoptosis in radiation-resistant HCC cells and suppresses radiation-induced epithelial-mesenchymal transition to overcome radioresistance [117]. Compounds like (-)-agelasidine A and prodigiosin upregulate UPR components (PERK, IRE1α) and pro-apoptotic proteins (CHOP, Bax, caspases) while downregulating anti-apoptotic Bcl-2, synergistically activating intrinsic and extrinsic apoptotic pathways—effects reversible by the ERS inhibitor 4-PBA [94, 118]. Other agents, including Annona muricata L and Xanthatin, induce ERS via the PERK-eIF2α-ATF4-CHOP axis, with Xanthatin specifically promoting ATF4 nuclear translocation to amplify CHOP-dependent apoptosis, an effect abolished by CHOP knockdown [119, 120].

Under therapeutic pressure, the interplay between ERS and autophagy becomes a critical determinant of resistance. Sorafenib induces ERS but concurrently activates protective autophagy via the PERK–ATF4–Beclin1 pathway to counteract apoptosis, while low-dose melatonin inhibits this axis to restore sorafenib sensitivity [121]. In 5-fluorouracil (5-FU)-resistant cells, konjac glucomannan reverses resistance by downregulating TLR4 to reactivate the PERK/ATF4/CHOP pathway [122]. The ATP citrate lyase inhibitor BMS-303141 triggers the p-eIF2α/ATF4/CHOP axis, synergizing with sorafenib to suppress tumor growth [123], whereas ^125^I brachytherapy combined with lobaplatin amplifies apoptosis via the same pathway [124]. Additionally, ubiquitin-specific peptidase 18 attenuates ERS-mediated apoptosis by inhibiting PERK phosphorylation and CHOP expression [125], whereas total flavonoids of Oldenlandia diffusa simultaneously induce apoptosis and autophagy via PERK-eIF2α-ATF4 activation [126].

ERS-related molecules also govern HCC metastasis and microenvironment remodeling. The histidine-rich calcium-binding protein enhances ERS adaptation through the PERK-eIF2α-ATF4-CHOP axis, fostering anoikis resistance and metastasis, with its expression correlating with tumor size and TNM stage [127]. ERS-associated gene signatures link to increased immune cell infiltration in HCC, where PPP1R16A may remodel the TME via the MIF/CD74+CXCR4 signaling axis [128]. Melatonin selectively blocks ATF6, suppressing COX-2 to augment ERS-induced apoptosis, while the lncRNA RMRP regulates PERK-mediated apoptosis, with its knockdown enhancing cell death [121].

### Glioblastoma (GBM)

Therapeutic resistance in GBM is closely associated with dysregulation of ERS and the UPR. Research demonstrates that various natural compounds and targeted agents induce GBM cell death while overcoming treatment resistance by modulating ERS/UPR pathways. Sulforaphane selectively triggers apoptosis in GBM cells through activation of the ATF4–CHOP axis [129], whereas the proteasome inhibitor marizomib upregulates ERS markers such as GRP78, IRE1α, and CHOP to induce caspase-3-dependent apoptosis, independent of ROS and autophagy [130]. Notably, remdesivir exerts antitumor effects via PERK-mediated UPR, demonstrating superior efficacy to temozolomide (TMZ) while maintaining a favorable safety profile [131].

The dynamic regulation of ERS pathways plays a dual role in GBM treatment resistance. TMZ-resistant cells exhibit suppressed ERS and enhanced proteasomal activity, with PSMC2 overexpression inhibiting pro-death autophagy via the JNK-Bcl-2-Beclin1 pathway—an effect reversible by PSMC2 targeting [132]. Additionally, therapy-induced senescent GBM cells rely on the PERK-ATF4-CHOP axis for survival, and PERK inhibition promotes apoptosis while delaying recurrence [133]. Integrin α3 silencing disrupts β1 subunit maturation, leading to immature β1 accumulation and ERS-dependent DR5 upregulation, resensitizing resistant cells to TRAIL [134]. Combination strategies targeting key ERS nodes show significant therapeutic potential. The UBA1 inhibitor TAK-243 induces irreversible UPR by disrupting protein ubiquitination, with efficacy correlating with GRP78 expression, while the GRP78 inhibitor HA15 synergistically enhances its effects [135]. The PDI inhibitor CCF642 amplifies TMZ efficacy through irreversible UPR, and its nanoformulation markedly suppresses tumor growth in vivo [136]. Furthermore, metabolic interventions such as nutrient deprivation effectively eliminate glioma stem cells via ERS-mitochondrial coupling pathways, highlighting the therapeutic value of microenvironment modulation [134].

### Lung cancer (LC)

The progression and therapeutic resistance of lung cancer are closely linked to dysregulated ERS within the TME. Studies reveal that ERO1A shapes an immunosuppressive TME by balancing IRE1α and PERK signaling, and its ablation enhances PD-1 blockade efficacy while promoting ICD [91]. Similarly, Derlin-3 drives M2 macrophage polarization via the Hrd1/p38/PRDM1 pathway, fostering immune suppression [137]. Photodynamic therapy, through ROS-dependent mechanisms, induces ERS and DNA damage, significantly boosting tumor immunogenicity [138].

ERS-related molecular markers hold prognostic value in lung cancer. Risk signatures based on key ERS-related genes accurately predict patient survival, with high-risk groups exhibiting reduced immune infiltration and diminished treatment sensitivity [139]. Among ERS-associated lncRNAs, RP11-295G20.2 promotes tumor progression by regulating cell migration and ERS [140]. TBL2, a novel driver gene, aids tumor adaptation to ERS by upregulating ATF4 and serves as an independent poor prognostic factor in LUAD [141]. Therapeutic strategies targeting the ERS-death axis demonstrate antitumor effects. Natural compounds like Icariside II [142] and Dihydroartemisinin [143] enhance chemosensitivity by activating ERS pathways, while the Gefitinib derivative L1Au2 induces ERS-dependent immunogenic death by dual-targeting TrxR and EGFR [144]. Narciclasine upregulates NOXA via the IRE1α-JNK/p38 axis, synergizing with cisplatin to amplify apoptosis [145]. Fascaplysin triggers ferroptosis and apoptosis through ROS-mediated ERS while upregulating PD-L1 to improve immunotherapy [146].

The ERS regulatory network intersects with metabolic reprogramming. 3,3’-Diselenodipropionic acid induces ERS-associated apoptosis via reductive stress, with confirmed in vivo antitumor activity [147]. The novel PPARɣ ligand PPZ023 provokes ERS-dependent death through the PPARγ/ROS/PERK axis, and its exosomes transmit death signals to overcome radioresistance [148].

### Pancreatic ductal adenocarcinoma (PDAC)

In PDAC, ERS drives disease progression and therapeutic resistance through multiple mechanisms within the TME. Single-cell analyzes reveal heterogeneity in tumor-associated neutrophils (TANs), with terminally differentiated TAN-1 subsets exhibiting enhanced glycolysis regulated by the transcription factor BHLHE40 [149]. These TANs foster an immunosuppressive TME via CCL5 secretion and upregulation of the immune checkpoint Nectin2, while targeted interventions can reverse T-cell exhaustion [150].

ERS plays a central role in PDAC treatment resistance. RUNX1 activates the BiP/PERK/eIF2α pathway to promote gemcitabine resistance, an effect reversible by RUNX1 inhibition [151]. Innovative nanodelivery systems, such as CB-5083/miR-142 co-loaded nanoparticles, induce immunogenic cell death by multitargeting ERS and immune checkpoints [152]. The GRP78 inhibitor YUM70-PROTAC triggers irreversible ERS-dependent apoptosis, demonstrating strong synergy [153]. Metabolic reprogramming intersects with ERS regulation. BZW1, a PERK adaptor protein, enhances glycolysis via HIF1α/c-Myc, and its suppression overcomes metabolic stress resistance [154]. Natural compounds like secoemestrin C disrupt ER proteostasis to induce aberrant YAP degradation, while 2-hydroxy nervonic acid provokes ERS-mediated apoptosis through metabolic derivatives [155].

Therapeutically, anlotinib activates ERS via the ROS-PERK/eIF2α/ATF4 axis, with Nrf2 inhibition augmenting efficacy [156]. Curcumin/gelatin-blended nanofibrous mats sustain ERS induction to suppress STAT3 signaling and promote apoptosis [157]. Multifunctional nanoplatforms amplify immunogenic death through ERS, synergizing powerfully with immunotherapy [158].

## Conclusions and future perspectives

Therapeutic strategies targeting the ERS-death axis through precise modulation of UPR core pathways (PERK, IRE1α, ATF6) and downstream molecules (CHOP, GRP78, XBP1, etc.) have demonstrated significant antitumor potential in preclinical studies. As comprehensively summarized in Small Molecule Compounds (Table 1) and Biomacromolecules, Natural Extracts, and Novel Formulations (Table 2), these preclinical evidences not only validate the therapeutic potential across diverse cancer types but also provide a roadmap for future drug development by systematically categorizing agents based on their specificity for UPR components and translational status.

**Table 1:** Small molecule compounds.

Classification	Agent	Cancer type	Mechanism of action	Clinical trial phase	Ref
PERK agonist	CCT020312	BC	Activates PERK/eIF2α/ATF4/CHOP pathway, mediating G1 cell cycle arrest and apoptosis.	Preclinical	[105, 106]
Cardiac glycoside	Oleandrin	BC	Induces ICD via PERK/eIF2α/ATF4/CHOP pathway.	Preclinical	[104]
Quinone derivative	Dihydrotanshinone I	BC	Targets ERp57, inducing ERS and UPR activation, leading to apoptosis.	Preclinical	[162]
Macrolide	I1amycin E	BC	Promotes apoptosis via ERS-CHOP-Bcl-2 pathway.	Preclinical	[163]
Synthetic compound	AMC-04	BC	Activates ATF4-CHOP and DR5 expression, promoting apoptosis.	Preclinical	[164]
Natural phenol	Curcumin	CRC	Stimulates ATF6 expression and nuclear translocation to activate ERS, influencing apoptosis.	Phase I/II (for cancer prevention/therapy)	[111]
Natural compound	Gambogenic acid	CRC	Induces ERS via Aurora A pathway.	Preclinical	[112]
Triterpenoid saponin	α-Hederin	CRC	Disrupts proteostasis by blocking ERAD and autophagic flux, leading to cell death.	Preclinical	[113]
Proteasome inhibitor	Carfilzomib	CRC	Induces UPR via enhanced CHOP expression and ATF6 activity, increasing caspase-3/7-mediated apoptosis.	Approved (for multiple myeloma); Phase II (for CRC)	[114]
Natural compound	Trichodermic acid	CRC	Induces autophagy, attenuating ERS-mediated apoptosis.	Preclinical	[116]
Flavonol	Fisetin	HCC	Increases apoptosis via intracellular Ca²⁺ release, PERK-ATF4-CHOP signaling, and GRP78 exosome induction.	Preclinical	[119]
Natural red pigment	Prodigiosin	HCC	Induces apoptosis via ERS.	Preclinical	[120]
Sesquiterpene lactone	Xanthatin	HCC	Promotes ATF4 activation, inducing ERS-mediated apoptosis.	Preclinical	[122]
Hormone + TKI	Melatonin + Sorafenib	HCC	Low melatonin inhibits autophagy via PERK-ATF4-Beclin1 pathway, increasing sensitivity to sorafenib.	Phase I/II (for combination therapy)	[123]
Chemotherapy + Polysaccharide	5-Fluorouracil + Konjac glucomannan	HCC	Downregulates TLR4, activating PERK/ATF4/CHOP signaling, enhancing apoptosis in 5-FU-resistant HCC cells.	Preclinical (for combination)	[124]
ATPase inhibitor	BMS-303141	HCC	Induces ERS and activates p-eIF2α/ATF4/CHOP axis to promote apoptosis; synergizes with sorafenib.	Preclinical	[125]
Platinum chemotherapeutic	Lobaplatin	HCC	Induces apoptosis and upregulates PERK-eIF2α-ATF4-CHOP pathway, suppressing proliferation.	**Approved** (in China for various cancers)	[126]
Isothiocyanate	Sulforaphane	GBM	Activates UPR via ATF4-CHOP axis, inducing apoptosis.	Phase I/II (for various cancers)	[131]
Proteasome inhibitor	Marizomib	GBM	Triggers ERS and promotes apoptosis.	Phase III (for GBM)	[132]
Nucleotide analog	Remdesivir	GBM	Enhances ERS and activates PERK-mediated UPR to induce apoptosis.	**Approved** (for COVID-19); Preclinical (for GBM)	[133]
Artemisinin derivative	Dihydroartemisinin	GBM	Induces mitochondrial/ER-mediated apoptosis and autophagic cell death.	Phase I/II (for various cancers)	[165]
Flavonoid	Icariside II	LC	Enhances cisplatin-induced cell death by promoting ERS signaling in NSCLC cells.	Preclinical	[144]
Artemisinin derivative	Dihydroartemisinin	LC	Triggers ICD via ferroptosis-induced ERS and DNA damage.	Phase I/II (for various cancers)	[145]
Gold complex	L1Au2	LC	Promotes GPX4 degradation, inducing ferroptosis, ERS, and ICD.	Preclinical	[146]
Alkaloid	Narciclasine	LC	Enhances cisplatin-induced apoptosis via UPR-mediated NOXA and MCL1 regulation.	Preclinical	[147]
Alkaloid	Fascaplysin	LC	Induces apoptosis and ferroptosis in vitro, improves anti-PD-1 immunotherapy sensitivity in vivo.	Preclinical	[148]
Selenorganic compound	3,3’-Diselenodipropionic acid	LC	Induces apoptosis via extrinsic/intrinsic pathways and ERS in a p53-independent manner.	Preclinical	[149]
Synthetic compound	PPZ023	LC	Causes cell death via PERK-eIF2α-CHOP axis in NSCLC cells/exosomes.	Preclinical	[150]
GRP78 inhibitor	YUM70	PDAC	Binds and inactivates GRP78, leading to ERS-mediated apoptosis.	Preclinical	[155]
Ester	Secoemestrin C	PDAC	Enhances ERS-triggered apoptosis via ERAD ubiquitination and YAP downregulation.	Preclinical	[157]
Multi-target TKI	Anlotinib	PDAC	Induces apoptosis via ROS accumulation-mediated ERS.	**Approved** (in China for lung cancer); Phase II (for PDAC)	[158]

**Table 2:** Biomacromolecules, natural extracts, and novel formulations.

Classification	Agent	Cancer type	Mechanism of action	Clinical trial phase	Ref
Secreted protein	SPARC	CRC	Interacts with GRP78, disrupting ERS signaling and enhancing cell death via PERK/eIF2α and IRE1α/XBP-1 pathways.	Preclinical	[115]
Deubiquitinase	Ubiquitin-specific peptidase 18 (USP18)	HCC	Attenuates ERS via PERK-eIF2α-ATF4 axis, reducing apoptosis.	Preclinical	[127]
Plant extract	Annona muricata L	HCC	Induces apoptosis via ERS.	Preclinical	[121]
Dietary fiber	Resistant starch	CRC	Enhances apoptosis via ERS-mediated mitochondrial apoptotic pathway.	Preclinical	[117]
Herbal extract	Total flavonoids of Oldenlandia diffusa (Willd.) Roxb.	HCC	Simultaneously induces apoptosis and autophagy by activating PERK-eIF2α-ATF4 pathway.	Preclinical	[128]
Photosensitizer-based therapy	MPPa-PDT	BC	Mediated through ERS-induced autophagy.	Preclinical	[166]
Nanofibrous mat	urcumin/gelatin-blended nanofibrous mat	PDAC	Increases ROS production and ERS-mediated apoptosis.	Preclinical	[159]

For the PERK pathway, inhibitors (e.g., GSK2656157) that block protective autophagy or agonists (e.g., CCT020312) that enhance pro-death signals can reverse resistance in non-small cell lung cancer and triple-negative breast cancer, respectively [17, 18]. IRE1α-targeting agents (e.g., STF-083010) suppress kinase or nuclease activity to induce apoptosis in pancreatic cancer while mitigating mitochondrial and autolysosomal alterations [159]. ATF6 modulators (e.g., curcumin and melatonin) selectively regulate the ERS-death axis in colorectal and liver cancers by activating or inhibiting its transcriptional function [20, 21]. Downstream targeting approaches—such as GRP78 inhibitors (HA15, YUM70) triggering irreversible ERS in pancreatic cancer [22, 23] and CHOP enhancers (e.g., oleandrin) inducing subtype-specific apoptosis in breast cancer [123]—highlight the advantages of precision modulation. Combination strategies further expand therapeutic potential: PERK inhibitors paired with mRNA vaccines remodel the immune microenvironment in glioblastoma [24]; nanocarrier-mediated ERS inducers synergize with PD-1 inhibitors to convert “cold” tumors to “hot” [37]; and metabolic interventions (e.g., 2-deoxyglucose] combined with autophagy inhibitors amplify lethal ERS effects in triple-negative breast cancer [25].

Despite these promising preclinical results, translating ERS-death axis targeting strategies into clinical practice faces several critical challenges. Tumor heterogeneity leads to markedly divergent ERS-death axis characteristics (e.g., differential responses between ER⁺ and triple-negative breast cancers) [15, 16, 26], complicating the establishment of unified treatment protocols. The double-edged nature of ERS modulation necessitates precise control over activation intensity to avoid toxicity in normal tissues. Current drug delivery systems still lack sufficient targeting specificity and tissue penetration, as exemplified by the fibrotic stroma in pancreatic cancer that impedes nanoparticle delivery [27, 28]. Additionally, complex resistance mechanisms (e.g., FLIP upregulation and exosomal miRNA-mediated immune evasion) [26] further hinder therapeutic efficacy.

To overcome these barriers, future research must focus on [160, 161]: utilizing multi-omics technologies to identify ERS-specific biomarkers for improved patient stratification; developing spatiotemporally controllable ERS modulation systems that balance pro-death effects with adaptive resistance; engineering intelligent nanocarriers with enhanced targeting capabilities; and exploring triplet combination strategies integrating ERS modulators, ICIs, and metabolic interventions for multidimensional synergy against resistance. Breakthroughs in these areas will facilitate the transition of ERS-death axis research from fundamental theory to personalized clinical applications, ultimately providing innovative approaches for precision cancer therapy.
